# Altered trunk and lower extremity movement coordination after neuromuscular training with and without external focus instruction: a randomized controlled trial

**DOI:** 10.1186/s13102-021-00326-9

**Published:** 2021-08-17

**Authors:** Bahram Sheikhi, Amir Letafatkar, Abbey C. Thomas, Kevin R. Ford

**Affiliations:** 1 Biomechanics and Corrective Exercise Laboratory, Faculty of Physical Education and Sports Sciences, Kharazmi University, Mirdamad Blvd., Hesari St, Tehran, Iran; 2 Department of Kinesiology, University of North Carolina at Charlotte, Charlotte, NC USA; 3 Department of Physical Therapy, Congdon School of Health Sciences, High Point University, One University Parkway, High Point, NC 27268 USA

**Keywords:** Segment coupling, Feedback, Anterior cruciate ligament, Drop vertical jump

## Abstract

**Background:**

This study sought to determine the effects of a 6-week neuromuscular training (NMT) and NMT plus external focus (NMT plus EF) programs on trunk and lower extremity inter-segmental movement coordination in active individuals at risk of injury.

**Methods:**

Forty-six active male athletes (controls = 15, NMT = 16, NMT plus EF = 15) participated (age = 23.26 ± 2.31 years) in this controlled, laboratory study. Three-dimensional kinematics were collected during a drop vertical jump (DVJ). A continuous relative phase (CRP) analysis quantified inter-segmental coordination of the: (1) thigh (flexion/extension)—shank (flexion/extension), (2) thigh (abduction/adduction)—shank (flexion/extension), (3) thigh (abduction/adduction)—trunk (flexion/extension), and (4) trunk (flexion/extension)—pelvis (posterior tilt/anterior tilt). Analysis of covariance compared biomechanical data between groups.

**Results:**

After 6 weeks, inter-segmental coordination patterns were significantly different between the NMT and NMT plus EF groups (*p* < 0.05). No significant differences were observed in CRP for trunk-pelvis coupling comparing between NMT and NMT plus EF groups (*p* = 0.134), while significant differences were observed CRP angle of the thigh-shank, thigh-trunk couplings (*p* < 0.05).

**Conclusions:**

Trunk and lower extremity movement coordination were more in-phase during DVJ in the NMT plus EF compared to NMT in active individuals at risk of anterior cruciate ligament injury.

*Trial registration*: The protocol was prospectively registered at UMIN_RCT website with ID number: UMIN000035050, Date of provisional registration 2018/11/27.

**Supplementary Information:**

The online version contains supplementary material available at 10.1186/s13102-021-00326-9.

## Background

Lower extremity musculoskeletal injuries, including anterior cruciate ligament (ACL) tears, frequently occur during sports [1, 2]. Considering the time-loss, financial expenses and detrimental influence on an athlete’s performance and general well-being, preventing such injuries should be prioritized [2]. Though the mechanisms underlying lower extremity injuries are multifactorial [3], many associated biomechanical and neuromuscular factors are modifiable [4, 5].

During activity, joints/segments are coupled such that movement of one influences action at adjacent joints/segments. During certain activities, the adjacent limbs have coupled movement and exhibit a unique coordination pattern [6, 7]. Coordination is a product of dynamic interactions between the nervous system, musculoskeletal system and environment, and an organized relationship between these constituents allows for flexibility in movement patterns [8]. Unfortunately, a significant change in coordination patterns or altered motor control may cause joint injuries [6].

For example, a reduction in proximal function may increase injury risk by inappropriately controlling distal joint/segment displacements [3]. In particular, inter-joint coordination represents the relationship between adjacent two joints, while inter-segment coordination is associated with one joint alone [9].

Coordination in the trunk and lower extremity has been measured using continuous relative phase (CRP), a method that derives the phase angle of a segment or joint from its position-velocity curve. Previous studies which have used CRP have used joint angles as the original signals [10–12]. However, the use of joint angles is contradictory to modeling the segments as pendula. Segment angles measured relative to an external reference frame allow meaningful and interpretable results that can be used to describe phase relationships properly from a dynamical systems framework perspective [11]. The difference between two-phase angles gives the CRP angle and is used as a measure of coordination [10]. In terms of relative phase, for the range (− 180°, 180°) a continuous relative phase value of 0° represents in-phase behavior and values of − 180° and 180° represent anti-phase behavior [11].

Lower extremity inter-segmental coupling is task dependent [13]. Coordination of trunk, pelvis, thigh, and shank segments has been investigated during functional tasks, including vertical jump, countermovement jump [14], drop vertical jump (DVJ) [15, 16], anticipated and unanticipated sidestepping [17], single-leg landing [18], walking and running [19] and cutting [20]. As many injuries occur during landing, coordination during jump landing is an important consideration for injury prevention [21].

Clinical identification of and focused interventions for groups at risk for ACL injuries are important to improve injury prevention. In an attempt to prevent injuries, many researchers have relied on the retention framework, neuromuscular training (NMT) and targeted muscle strengthening exercises to analyze and correct aberrant patterns such as dynamic knee valgus [22–24]. Despite the best efforts of these programs, injuries continue to occur. Benjaminse et al. [23] have suggested that incorporating feedback into NMT exercises may superiorly prevent injuries compared to NMT alone because feedback may accelerate learning and enhance retention and transfer of motor skills, including the pelvis, thigh, and shank alignment in suitable landing positions. Feedback exercises provide the subjects with these benefits through automated progress, which is comparable to motor plans or central adaptation [23]. Results of previous injury prevention programs incorporating external focus (EF) support the notion that EF is beneficial [22, 25]. In the EF, attention is directed towards the effect of the movement. EF enhances motor performance and technique and improves neuromuscular coordination. This is illustrated by greater knee flexion angles, decreased knee valgus, more center of mass displacement, and lower peak vertical ground reaction force. Importantly, EF improves retention of learned movement patterns compared to internal focus of attention feedback [23, 26]. These results all suggest being beneficial in reducing the risk of ACL injury [23, 27]. While the effects of NMT and EF on lower extremity biomechanics have been investigated [22, 28–30], further investigations are needed to explore the relationship between injury prevention programs and their effects on the movement coordination of different segments. It remains unknown, however, what influence NMT and NMT plus EF have on the coordination of the trunk, pelvis, thigh, and shank segments in active individuals. Thus, this study sought to determine inter-segmental coordination before and after NMT and NMT plus EF compared to control. We hypothesized that adding EF during NMT would yield more in-phase trunk and lower extremity movement coordination in active individuals at risk of ACL injury.

## Methods

### Participants

A priori power analysis (G*Power©, version 3.1, University of Dusseldorf, Dusseldorf, Germany) to obtain 80% statistical power with α of 0.05, and a medium effect size of 0.25, determined we would need 14 participants per group (total sample size of 42 participants). Allowing a dropout rate of 10% and improved final statistical power, we enrolled 16 participants per group (total of 48 participants). This effect size was comparable to previous research reporting changes in hip and knee biomechanics after training [31].

Forty-eight competitive male handball, volleyball, and basketball players were randomly assigned by using the website http://randomizer.org/ (Social Psychology Network, Connecticut, USA), into one of three groups as follows: NMT plus EF group (n = 16), NMT (n = 16) or control group (n = 16). Concealed allocation was performed using a computer-generated block randomized table of numbers (1; control group, 2; NMT group, and 3 for NMT plus EF group). The random numerical sequence was placed in sealed opaque envelopes in a box. Another researcher (blinded to the baseline assessment) opened envelopes and proceeded with training according to the group assignment. Prior to data collection, this study was approved by the research ethics committee of the Tarbiat Modares university of medical sciences (Approval ID: IR.MODARES.REC.1397.117). The protocol was prospectively registered at UMIN_RCT website (ID number: UMIN000035050). All participants were informed of the study procedures, and they signed an informed consent form prior to participating, in accordance with the Declaration of Helsinki.

**Fig. 1:**
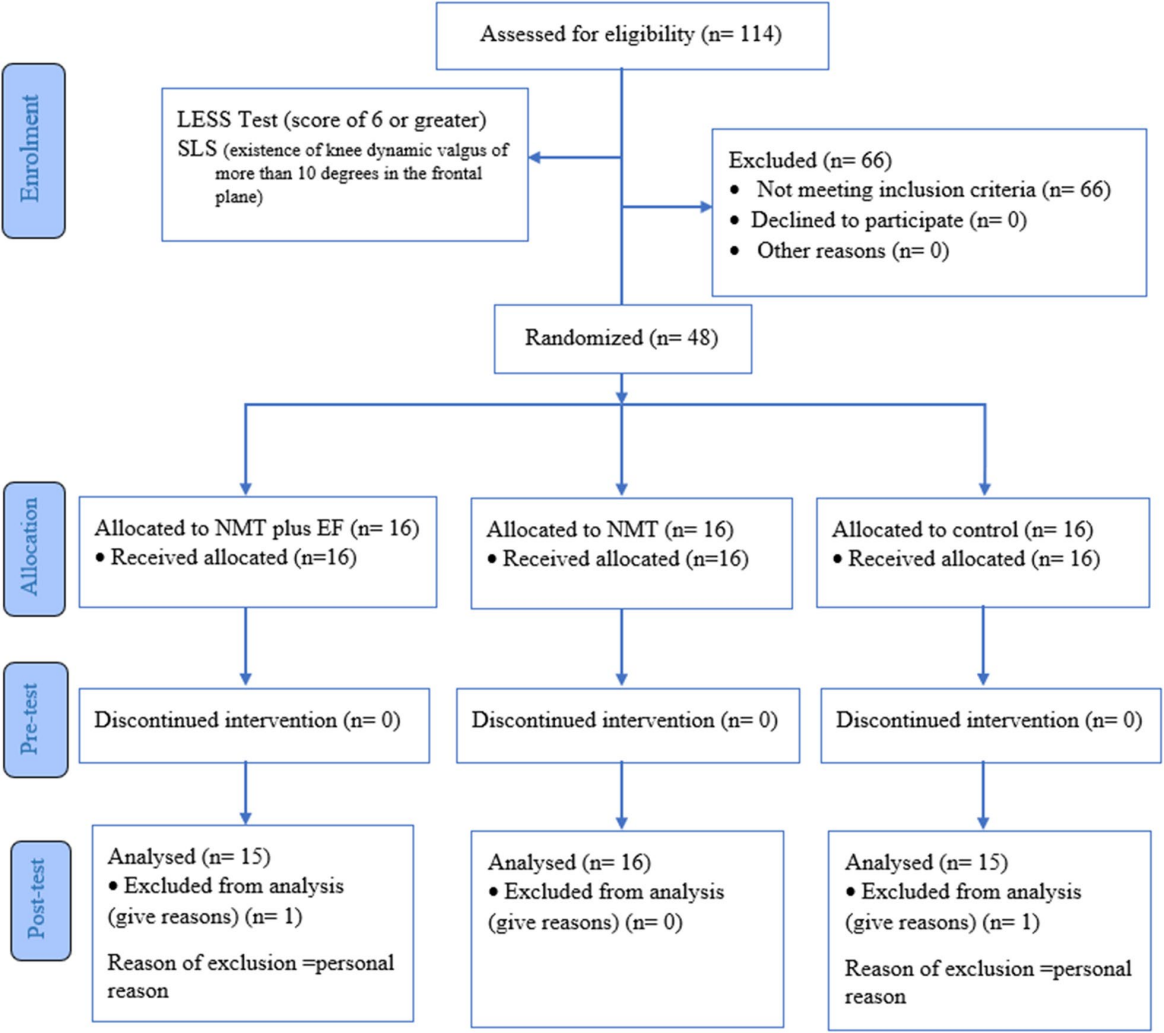
Consort flow diagram

Two participants were lost to follow-up due to personal reasons (control group, n = 1; NMT plus EF group, n = 1). No undesirable or adverse events were reported. Participants who did not complete the follow-up were excluded (Fig. 1).

To be eligible to participate, each participant was required to meet the following criteria: no history of surgery in the lower extremity in the previous 6 months, no history of non-corrected neurological, vestibular, visual and/or hearing impairments, no musculoskeletal injury that could interfere with or contraindicate the assessment procedures and normal body mass index (BMI; 19 to 25 kg/m^2^). Additionally, score of 6 or greater on the landing error scoring system (LESS) [32] and observable dynamic knee valgus was defined as a ≥ 10° frontal plane knee angle increase, as determined by visual observation, during the descent phase of the majority of the observed single leg squat trials [33]. Potential participants were excluded if they reported any current pain, history of any trunk and lower extremity injury or surgery within the past 6 months [30]. Participants did not report any injury that would have impeded their training or influenced their maximal physical performance.

### Procedures

Prior to the tests, the participants executed a standardized warm-up protocol consisting of a series of double-leg squats (2 × 8 repetitions) and double leg maximum jumps (2 × 5 repetitions), followed by calf-stretching with a straight and bent knee. All participants were asked to refrain from training, maintain a regular diet, and avoid smoking, caffeine and alcohol for 24 h prior to testing sessions. All participants were evaluated at the same time of the day in a laboratory where the ambient temperature was 24 °C.

Three-dimensional kinematics data were collected during DVJ. For the DVJ, participants dropped from a 31 cm high box to a distance of 50% of their height away from the box, and immediately perform a maximal vertical jump to reach an overhead target. The target height was set to their pre-recorded maximal vertical jump height. This task was performed three times with a 1-min rest between repetitions to minimize fatigue [15, 16].

**Fig. 2:**
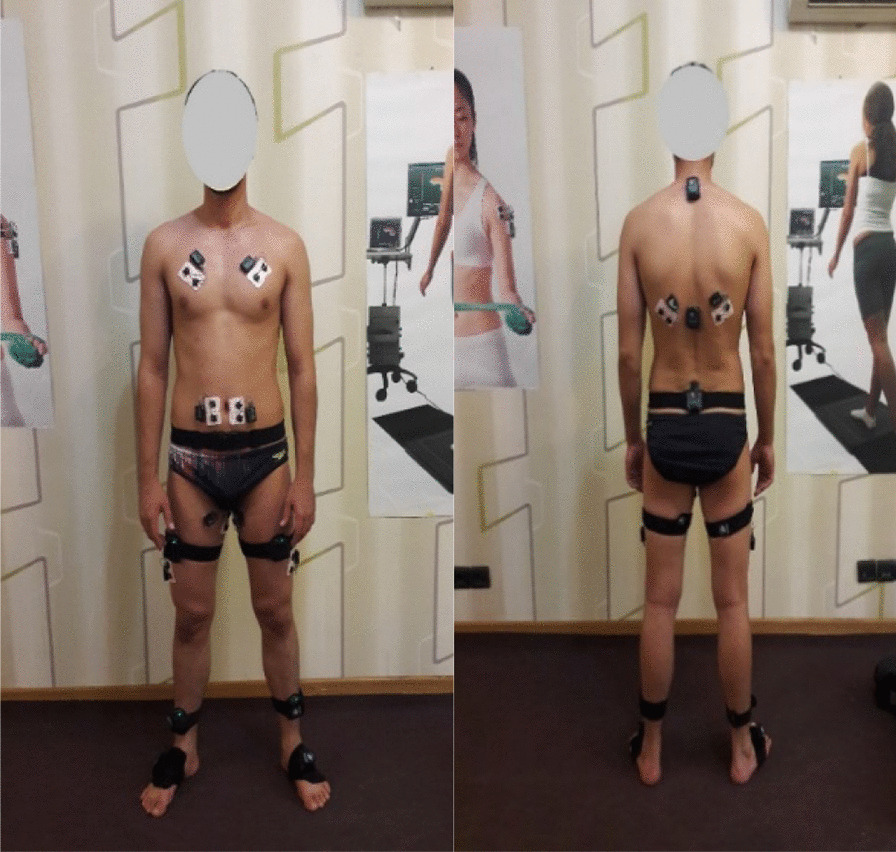
Sites of wireless sensors for kinematic data collection

A three-dimensional motion-tracking system (MyoMotion; Noraxon Inc., Scottsdale, AZ, USA) sampling at 200 Hz was used to analyze the kinematic variables. The accuracy and reliability of MyoMotion system were reported by Schmitz et al. [34]. Myo-Motion Research inertial sensors were placed according to the rigid-body model, with 9 segments defined in the MR3 software as shown in Fig. 2. Sensors were placed to the surface of the 7th cervical spinous process (upper thoracic), the 12th thoracic spinous process (lower thoracic), at the midpoint of the posterior superior iliac spine, the frontal attachment on lower quadrant of quadriceps, slightly above the patella, shanks (frontal on the tibia), and top of the upper foot, slightly below the ankle. The sensors were attached bilaterally with pelvis fixation straps and elastic straps. Prior to the movement trials, an initial standing calibration trial was conducted. All the intended data were assessed at baseline and after the interventions.

Following baseline testing, NMT occurred 3x/week for 6 weeks (18 sessions total). Each session lasted approximately 45 min. The NMT protocols were based on the protocol introduced by Di Stasi et al. [35] (see Additional file 1: Appendices A–K**)**. Briefly, participants in NMT plus EF group received 6 EF sessions [23] followed by 12 NMT sessions (see Additional file 1: Appendices L–N). All interventions were performed under supervision by an experienced athletic trainer in health center and human performance laboratory of Kharazmi University. These programs were chosen because they may successfully reduce the risk of ACL injury in athletes. The control group continued its typical training during the 6 weeks period, which focused on improving sport related techniques and skills.

### Data reduction and analysis

Kinematics data were filtered using a fourth-order Butterworth low-pass filter with a 6 Hz cut-off frequency. A CRP was used to quantify coordination of the trunk, pelvis, thigh and shank segments [10]. Before calculating CRP, angular displacement and angular velocity were calculated for the trunk, pelvis, thigh and shank. The following intra- segmental couplings during the landing phase of the DVJ were examined: (1) thigh (flexion/extension)—shank (flexion/extension), (2) thigh (abduction/adduction)—shank (flexion/extension), (3) thigh (abduction/adduction)—trunk (flexion/extension), and (4) trunk (flexion/extension)—pelvis (posterior tilt/anterior tilt). Landing was defined as initial contact with the force platform to the lowest point of the center of mass. The phase plot was obtained by plotting angular position on the x-axis against angular velocity on the y-axis for each segment. In order to calculate phase angle, phase plots were normalised for each trial according to Eqs. (1) and (2), respectively.

1 $${\text{Horizontal axis}}:\theta_{i} = \frac{{2 \times \left[ {\theta_{i} - {\text{min}}\left( {\theta_{i} } \right)} \right]}}{{{\text{max}}\left( {\theta_{i} } \right) - {\text{min}}\left( {\theta_{i} } \right)}}$$

2 $${\text{Vertical axis}}:\omega_{i} = \frac{{\omega_{i} }}{{{\text{max}}\left[ {{\text{max}}\left( {\omega_{i} } \right)\max \left( {\omega_{i} } \right)} \right]}}$$

where θ is the segment angle, **i** the data point within ground contact and ω is the segment angular velocity. The phase angle was determined as the four-quadrant arctangent angle relative to the right horizontal axis and a line drawn from the origin to a specific data point ($\emptyset ,\omega$) according to Eq. 3:

3 $$\emptyset_{i} = tan^{ - 1} \left( {{\raise0.7ex\hbox{$\omega $} \!\mathord{\left/ {\vphantom {\omega \theta }}\right.\kern-\nulldelimiterspace} \!\lower0.7ex\hbox{$\theta $}}} \right)$$

Finally, in each coupling, the distal segment was subtracted from the proximal segment according to Eq. (4) and used as the coordination of two segments.

4 $$CRP_{i} = \emptyset_{i}^{distal} - \emptyset_{i}^{proximal }$$

The CRP ranged between − 180° and 180°. A CRP close to 0° meant that the two segments evolve “in-phase”, while a CRP close to 180° or − 180° corresponded to an “anti-phase”. Finally, a positive CRP indicates that the proximal segment leads the distal segment. A negative CRP indicates that the distal segment leads the proximal segment. All data were processed using custom MATLAB scripts (version 8.4, 2014b, Mathworks, Natick, MA).

### Statistical analysis

The Shapiro–Wilk and Levene’s tests were conducted to evaluate the normality and homogeneity assumptions, and all the tested variables showed *p* > 0.05. Group demographics were compared using one-way analysis of variance (ANOVA). The difference between groups (NMT plus EF, NMT, Control) were compared using analysis of covariance (ANCOVA). Percentage changes (%) from pre to post-training were calculated. Effect sizes (ES) using partial eta squared (*η*$$\begin{array}{c}2\\ p\end{array}$$) were calculated. ES were classified as small (0.01), moderate (0.06), and large (0.14) [36]. Statistical significance was set a priori at ≤ 0.05. All data analyses were conducted on SPSS software version 26 (IBM Corp., Armonk, NY, USA).

## Results

At baseline, there were no significant differences in demographic characteristics between the three intervention groups (Table 1). The compliance with training was high; with the mean attendance rate at 95.8% for the NMT plus EF group, at 93.75% for the control group and at 100% for the NMT group.

**Table 1:** Demographic variables for NMT plus EF, NMT and control groups

Factors	NMT plus EF^a^ (n = 15)	NMT^b^ (n = 16)	Control (n = 15)	*P* value^c^
Age (year)	23.3 ± 2.3	23.1 ± 2.4	23.4 ± 2.4	0.92
Height (cm)	186.7 ± 7.3	185.4 ± 6.7	184.5 ± 5.8	0.66
Mass (kg)	76.9 ± 6.3	76.3 ± 5.9	76.5 ± 5.7	0.96
BMI (kg/m^2^)	22.1 ± 0.5	22.2 ± 0.7	22.5 ± 0.6	0.18
Breakdown of number of athletes from each sport, n (%)				
Basketball	5 (33)	4 (27)	6 (40)	NA
Volleyball	6 (32)	7 (37)	6 (32)	NA
Handball	4 (33)	5 (42)	3 (25)	NA

*BMI* body mass index, *NA* not applicable

^a^Neuromuscular training plus external focus

^b^Neuromuscular training

^c^*P* value of ANOVA

Analysis of CRP values of thigh (flexion/extension)-shank (flexion/extension) coupling were significantly different between groups (*F* = 13.48, *p* < 0.001, *η*$$\begin{array}{c}2\\ p\end{array}$$ = 0.391). Following training, thigh (flexion/extension)-shank (flexion/extension) coupling was more in-phase in the NMT plus EF and NMT groups (*p* < 0.05). In addition, significant differences were observed between NMT plus EF and NMT groups (mean difference = 3.71, *p* = 0.037). The negative value indicates a more proximal segment contribution to the coupled motion (Table 2). No significant changes were observed for the control group (*p* > 0.05).

**Table 2:** Mean coupling angles (SD) between thigh-shank, thigh-trunk and trunk-pelvis during DVJ

Variables (coupling)	Group	Baseline Mean ± SD	Six weeks Mean ± SD	Δ relative to baseline (%)	ES (*η*^2^_p_)^b^	*P* value^a^, *F*
Thigh (flexion/extension)—Shank (flexion/extension) (°)	NMT plus EF	− 26.95 ± 9.62	− 19.12 ± 7.74 ^‡d^	29.05 ↑	0.391^e^	< 0.001, 13.48
	NMT	− 26.57 ± 9.08	− 22.56 ± 7.23 ^‡^	15.09 ↑		
	Control	− 27.35 ± 7.84	− 26.90 ± 7.72	1.65 ↑		
Thigh (abduction/adduction)—Shank (flexion/extension) (°)	NMT plus EF	23.27 ± 9.20	11.34 ± 7.46 ^‡d^	51.27 ↓	0.484^e^	< 0.001, 19.73
	NMT	23.47 ± 9.42	17.40 ± 6.91 ^‡^	25.86 ↓		
	Control	22.13 ± 8.21	25.33 ± 5.44	14.46 ↑		
Thigh (abduction/adduction)—Trunk (flexion/extension) (°)	NMT plus EF	29.84 ± 11.09	19.30 ± 9.59 ^‡d^	35.32 ↓	0.441^e^	< 0.001, 16.58
	NMT	34.59 ± 12.08	28.07 ± 9.57 ^‡^	18.85 ↓		
	Control	30.72 ± 12.51	31.18 ± 11.61	1.50 ↑		
Trunk (flexion/extension)—pelvis (posterior tilt/anterior tilt) (°)	NMT plus EF	19.20 ± 7.80	9.41 ± 5.18 ^‡^	50.99 ↓	0.500^e^	< 0.001, 21.03
	NMT	18.10 ± 7.56	11.65 ± 6.82^c^	35.64 ↓		
	Control	17.21 ± 6.36	17.39 ± 6.80	1.05↑		

*NMT plus EF* neuromuscular training plus external focus, *NMT* neuromuscular training, *Δ* percent change (↓ decrease, ↑ increase)

^a^*P* value of ANCOVA

^b^Effect size

^c^Denotes significantly different than control group (*P* < 0.05)

^d^Denotes significantly different than NMT group (*P* < 0.05)

^e^Large effect size (0.14) based on the study of Cohen (1992)

During DVJ, significant differences were also observed between groups in the thigh (abduction/adduction)-shank (flexion/extension) coupling (*F* = 19.73, *p* < 0.001, *η*$$\begin{array}{c}2\\ p\end{array}$$ = 0.484). Thigh-trunk coupling was more in-phase in the NMT plus EF and NMT groups (*p* < 0.05). Also, significant differences were observed between NMT plus EF and NMT groups (mean difference = -6, *p* = 0.032) (Table 2).

The CRP values of thigh (abduction/adduction)-trunk (flexion/extension) coupling were significantly different between groups (*F* = 16.58, *p* < 0.001, *η*$$\begin{array}{c}2\\ p\end{array}$$ = 0.441). Following training, analysis of CRP showed that thigh–trunk coupling was more in-phase in the NMT plus EF and NMT groups (*p* < 0.05). Additionally, significant differences were observed between NMT plus EF and NMT groups (mean difference = − 5.25, *p* = 0.030). This result indicated that thigh (distal segment) motion relative to the trunk (proximal segment) motion was greater (Table 2).

A significant difference across groups was observed for trunk (flexion/extension)-pelvis (posterior tilt/anterior tilt) coupling (*F* = 21.03, *p* < 0.001, *η*$$\begin{array}{c}2\\ p\end{array}\hspace{0.17em}$$= 0.5). Trunk-pelvis coupling was more in-phase in the NMT plus EF and NMT groups (*p* < 0.05). There were no significant differences in trunk-pelvis coupling between the NMT plus EF and NMT groups (mean difference = − 2.98, *p* = 0.134) at the end of the study (Table 2).

## Discussion

The aim of this study was to investigate the effects of NMT plus EF and NMT on inter-segmental coordination of the trunk, pelvis, thigh, and shank during a DVJ in healthy male athletes at risk of ACL injury. Based on our results following training, the NMT and NMT plus EF groups demonstrated more in-phase CRP angle of thigh flexion/extension-shank flexion/extension, thigh abduction/adduction-shank flexion/extension, thigh abduction/adduction-trunk flexion/extension and trunk flexion/extension-pelvis posterior tilt/anterior tilt couplings. Furthermore, the NMT plus EF CRP angles for thigh-shank and thigh-trunk couplings were more in-phase compared to NMT.

Coordination may be described as the ability to reduce joint loading during movement through improved dynamic stability during landing tasks [21, 37]. Therefore, any increases in knee stability during landing may be the result of improved coordination, and any deviation from the normal coordination may provide evidence of dynamic stability impairment [21, 37]. During landing, hip and knee coordination generally determines dynamic stability of the leg. In the thigh to shank coupling, increasing shank movements relative to thigh movements may indicate a lower hip dynamic stability. Adaptations in patterns of segmental coordination have the potential to alter joint loading during dynamic activities, and therefore may also be associated with the development of lower extremity pathologies [38]. The anti-phase coordination pattern of lower extremity may result in increased knee joint stress [38].

The present research results showed that the average CRP of thigh (flexion/extension) to shank (flexion/extension) tended negatively towards the anti-phase. This pattern shows that, as the intensity of the activity increase, the femur follows the tibia attempting to reduce demands on the rotary knee stabilizers [13]. Compared with the shank segment, it can be argued that the thigh segment is of a larger CRP angle in this coupling because these segments are in the closed kinetic chain [14]. Having performed NMT and NMT plus EF, the CRP moves towards the in-phase. This factor may result from the proximal to distal muscle sequencing activation along the kinetic chain [39]. The kinetic chain efficiency is dependent on the activation sequencing of the muscles from proximal to distal [39]. A possible explanation for such results is that NMT facilitates adaptations focused on joint stabilization and safe movement patterns [40]. Also, neuromuscular control of the trunk, hip and knee is based on feedback control [35]. An EF has been found to result in accelerates the learning process or shortens the first stages of learning by facilitating movement automaticity (“constrained action hypothesis”). According to the constrained action hypothesis trying to consciously control one’s movements constrains the motor system by interfering with automatic motor control processes that would normally regulate the movement. On the other hand, focusing on the movement effect might allow the motor system to more naturally self-organize, unconstrained by the interference caused by conscious control attempts resulting in more effective performance and learning [23, 26]. Adding EF to NMT makes more in-phase coordination by unconstraining the motor system to optimize movement patterns [26]. Therefore, after performing prevention training, the knee coordinative response would be expected to change [21]. EF, with even minimal training may be effective in order to improve knee abduction displacement, a key kinematic variable that reduces injury risk [41].

Given the concept that healthy parts of a functioning motor system adapt their performance to compensate disrupting the vulnerable locations, it is expected that individuals susceptible to injury, or those currently injured, can exhibit more coordinated in-phase patterns as compared to healthy individuals [42]. According to Gribbin et al. [43] those with ACL injuries have knee joint coupling angles larger than their healthy counterparts, which it is interpreted as a reduction in hip relative to the knee movement. Since the knee is extending at the initial contact, its internal and external rotation is not high. Therefore, the hip should help move the frontal plane in this coupling. Davis et al. [44] argued that the proximal coordination pattern indicated more proximal joint (the hip) contribution to motion relative to the distal joint (the knee). In other words, if the coupling angle falls within the proximal phase range, the hip has a larger movement than the knee. In the coupling of the hip to the knee, the hip acts as a stabilizer in response to increased knee movement or instability. Therefore, the coupling angles increase so that the hip has fewer movements, and an attempt to increase the stability is shown [44]. The results obtained in this study differed from those of Davis et al. [44] and maybe the reason for this difference is the type of task being evaluated. It should not be overlooked that their study evaluated the coordination pattern in ACL reconstruction patients during gait, whereas the current study investigated coordination in healthy, active participants at high risk of ACL injury during DVJ.

Gheller et al. [14] argued that the thigh-to-trunk coupling in the countermovement-jump is less than 90° in the flexion phase. As relative to the low knee flexion angles (greater than 90°), the squat jump in the phase less than 70° is more in-phase. In the present study, the thigh-to-trunk coupling in following NMT plus EF (35.32%) and NMT (18.85%) was more in-phase relative to the baseline. As a result, it appears that athletes may benefit more from completing an NMT plus EF. Considering the kinetic chain, knee injuries during landing are generally associated with reduced neuromuscular control of the trunk, pelvis, and thigh set, which can mostly lead to changes in hip and trunk [39]. The position and load of segments is used to modify the descending movement commands. Regardless of force production, different accelerations are generated in the initial landing position due to the time modulation to generate force; hence, the coordination of movements changes. In this way, the lower velocity seems to provide a better synchronization between these segments, while a higher velocity is certainly associated with a greater instability in the coupling of thighs to trunk. Researchers have suggested that when the joints produce lower angular velocities, the knees and pelvis are more synchronous, indicating that there was more stability in the movement pattern. In this regard, it can be said that better synchronism between segments would be obtained during the jumps performed from a preferred position that is a usual movement pattern, and possibly already consolidated by the motor system [14, 39].

This study had several limitations. To our knowledge, this was the first study to report trunk and lower extremity coordination during landing before and after NMT. Therefore, comparing our results with existing literature was difficult. Further, this study enrolled healthy, active males at high risk of ACL injury that LESS and SLS tests were used to screen participants. Therefore, the results cannot be extrapolated to other populations, including females, sedentary individuals, and patients after musculoskeletal injury. Additionally, participants completed 6-weeks of NMT or NMT plus EF. Further research should determine the effects of different exercises with longer durations on movement coordination during functional tasks. Finally, only 3 trials were included in the CRP analysis. It could be suggested that additional trials are necessary to represent typical coordination during DVJ. The number of trials was selected based on previous research [14, 45, 46] demonstrating adequate statistical power (1-beta of 0.80) using 3 trials [47].

## Conclusion

NMT facilitates coordination of the trunk, pelvis, thigh, and shank segments during landing. When EF was applied to the NMT, the coordination improved over NMT alone. Thus, adding EF to NMT can help more in-phase inter-segmental coordination and reduce ACL injury risk.

## Supplementary Information

**Additional file 1**. Appendix 1. The neuromuscular training protocols.

## Data Availability

The datasets used and/or analysed during the current study are available from the corresponding author on reasonable request.

## Abbreviations

ACL: Anterior cruciate ligament
DVJ: Drop vertical jump
CRP: Continuous relative phase
NMT: Neuromuscular training
EF: External focus
ANOVA: One-way analysis of variance
ANCOVA: Analysis of covariance
ES: Effect size
