# Supplementary material for: Altered trunk and lower extremity movement coordination after neuromuscular training with and without external focus instruction: a randomized controlled trial
Source: BMC Sports Sci Med Rehabil. 2021 Aug 17;13:92. doi: 10.1186/s13102-021-00326-9 (PMC8369650; doi:10.1186/s13102-021-00326-9)
Supplement: Supplementary file 1 — Additional file 1. Appendix 1. The neuromuscular training protocols. [file 13102_2021_326_MOESM1_ESM.pdf]

Appendix A: Single-Leg Anterior Progression,

The trainer instructs the athlete to descend into a deep knee flexion hold upon each take-off and landing, avoiding excessive non-sagittal plane motion of the lower extremities and trunk. Phase 1 focuses on symmetry during take-off and landing, and the trainer should encourage jumping farther once the athlete has mastered the basic technique. Progression to phase 2 should occur only after the athlete can demonstrate proper technique during phase 1. Single-leg jumping for distance with proper take-o ff and landing is the focus of phase 3, prior to repeated anterior jumps in phase 4.

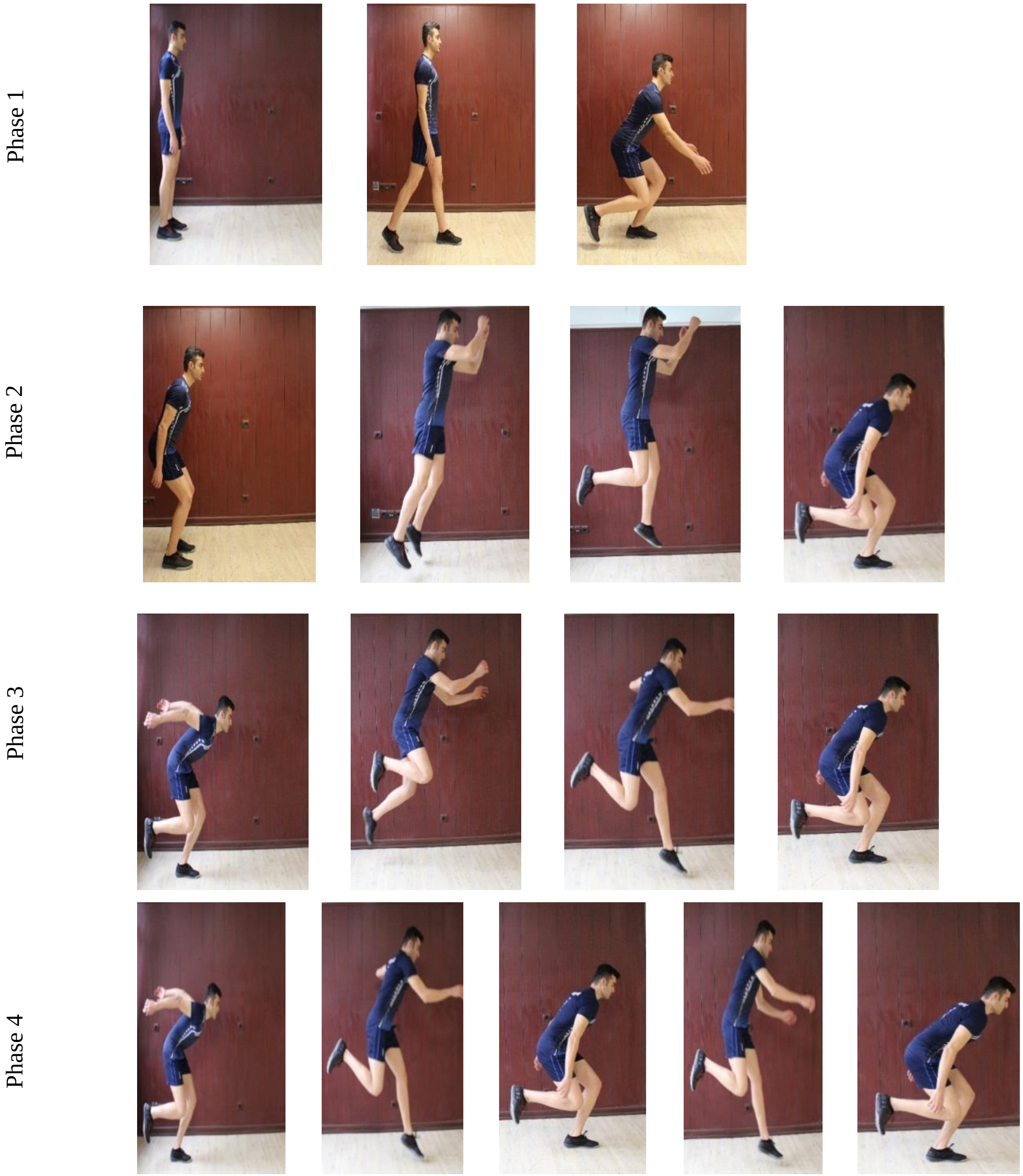

Appendix B: Single-Leg Lateral Progression

The trainer instructs the athlete to begin and end each hop hold with deep knee flexion, avoiding excessive non-sagittal plane motion of the lower extremities and trunk during take-o ff and landing. In the later phases, the athlete should also be instructed to minimize the amount of rebound (or reverberation) of the BOSU under the foot. Phase 4 should incorporate lateral and medial jumping.

Phase 1

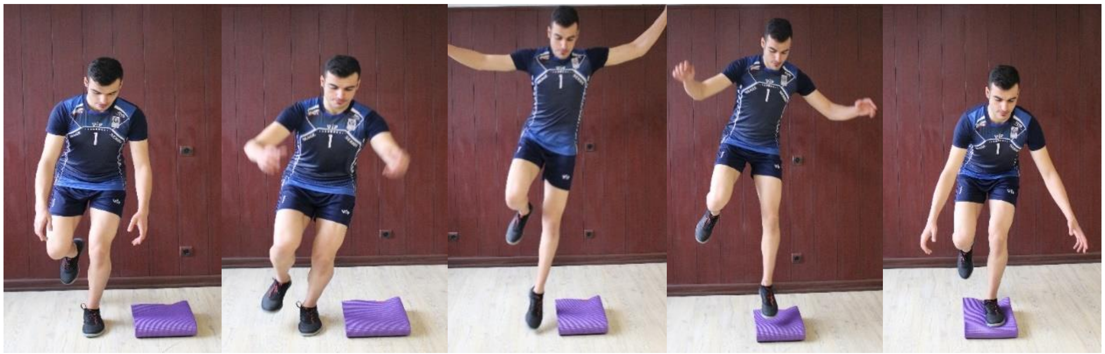

Phase 2

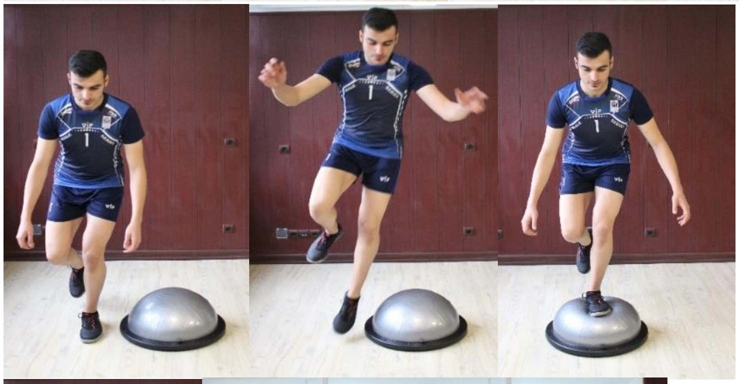

Phase 3

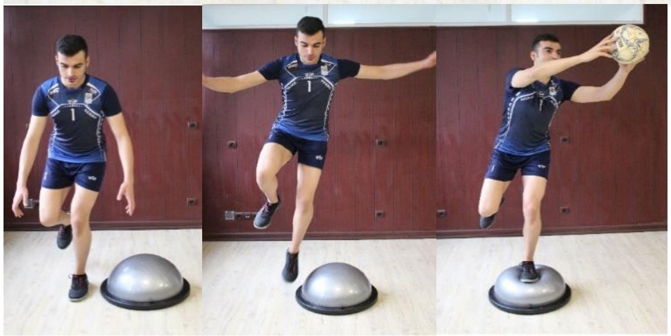

Phase 4

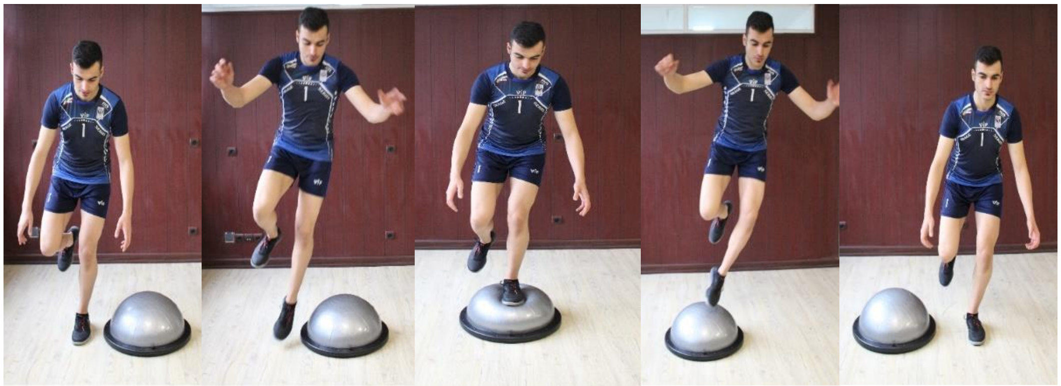

Appendix C: Lunge Progression

The trainer instructs the athlete to maintain most of the weight on the lead leg as they lunge forward into a deep knee extension, avoiding hyperextension of the trunk. A slight forward lean is acceptable, as this will assist the individual to drive off the lead leg. The athlete's knee should never advance beyond the ankle during the exercise. The clinician should also cue the athlete to avoid pausing between the lunge and upright portions of the task.

Phase 1

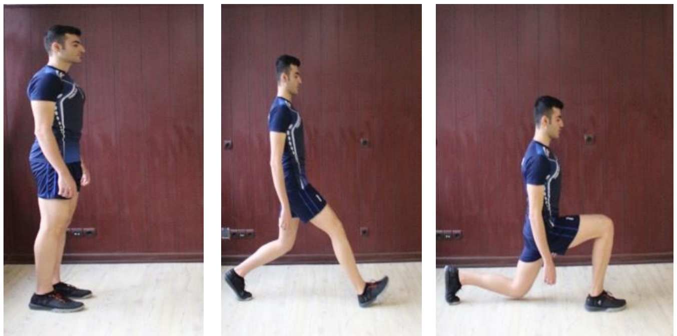

Phase 2

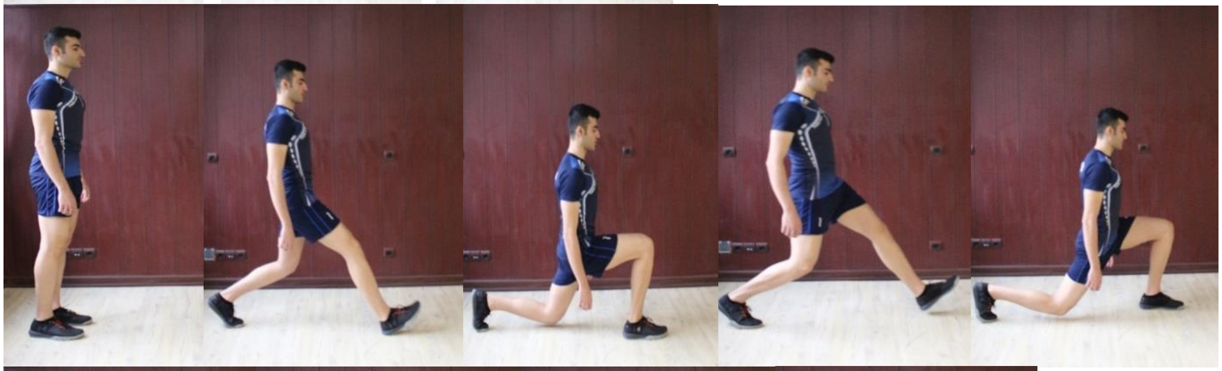

Phase 3

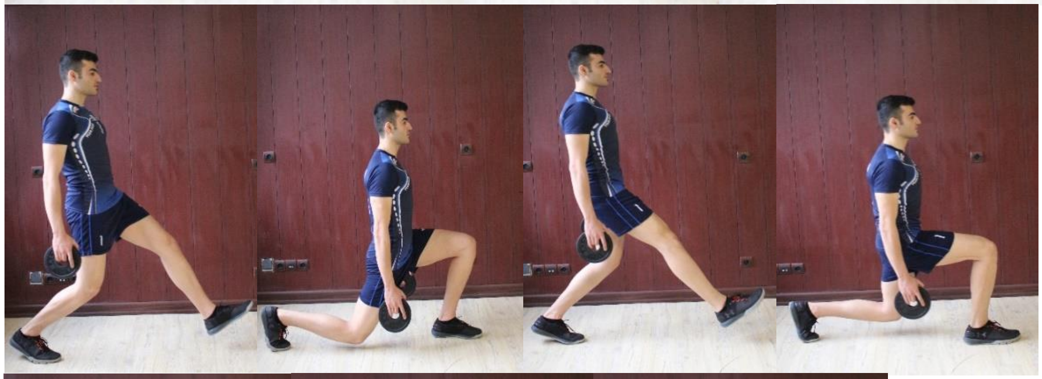

Phase 4

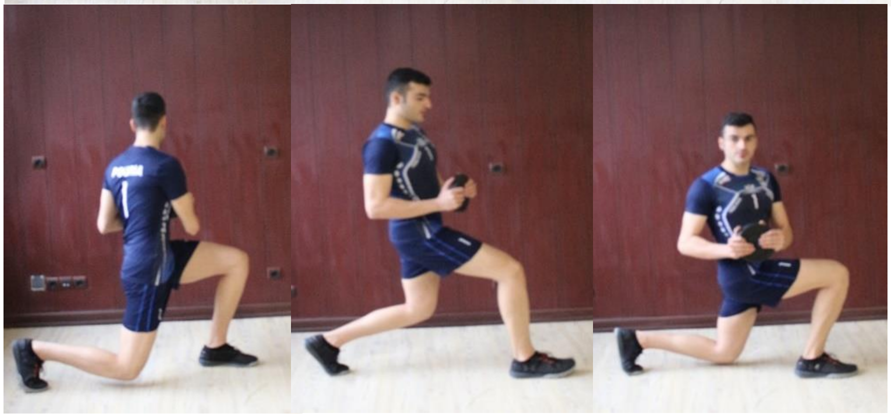

Appendix D: Tuck Jump Progression

The trainer instructs the athlete on the proper countermovement preparation (slight crouch downward, extending arms behind body) prior to the vertical jump. The vertical jump begins as the athlete vigorously swings the arms forward as they jump straight up, pulling their knees up as high as possible. The goal is to achieve a parallel position of both thighs in relation to the floor and to use a toe-to-midfoot rocker landing upon descent into a deep–knee flexion hold. As the athlete progresses from 2 consecutive jumps (phase 2) with proper technique to multiple consecutive jumps (phase 3), the clinician instructs the athlete to avoid excessive non–sagittal plane motion of the lower extremities and trunk, and to try to take off and land in the same footprint in which the task started. Tuck jumps performed over an object should be completed only if the athlete completes repeated phase 3 jumps with proper technique.

Phase 1

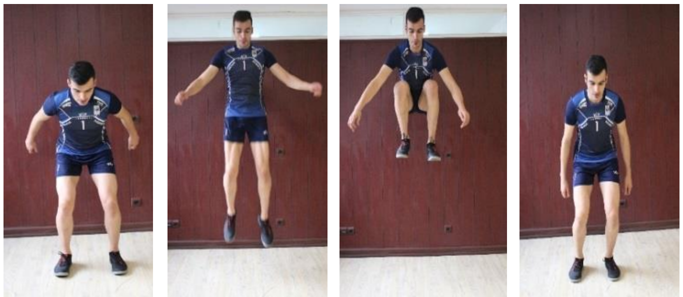

Phase 2

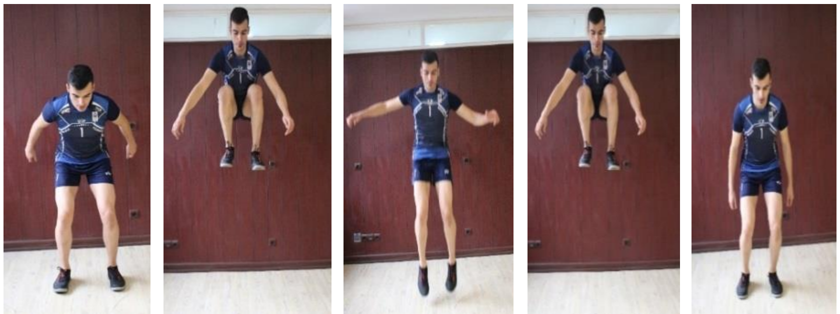

Phase 3

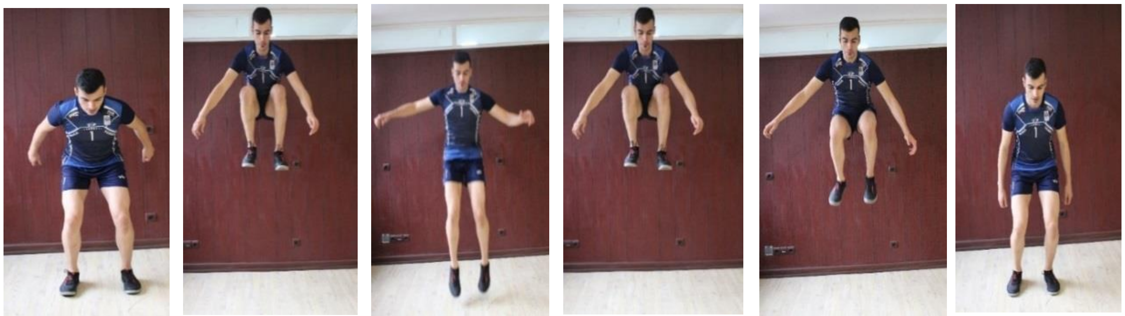

Phase 4

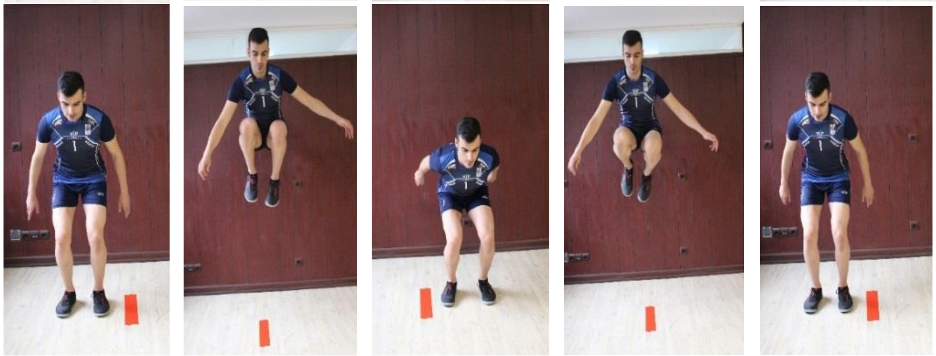

Appendix E: Lateral Jump Progression

The goal of this exercise is to focus on minimizing the frontal plane motion of the trunk and lower extremities during lateral jumping. The height of the jump is not the focus; rather, increasing speed with good technique is the criterion by which the athlete will be progressed to the next phase. A deep– knee flexion position is emphasized upon each take-off and landing, regardless of phase. The clinician should encourage the athlete to jump “close to the line” in preparation for quicker lateral movements. This exercise is progressed from double leg (phases 1 and 2) to single leg (phases 3 and 4) once the athlete can demonstrate symmetrical timing and proper alignment with single- (phase 1) and then repeated double-leg landing (phase 2).

Phase 1

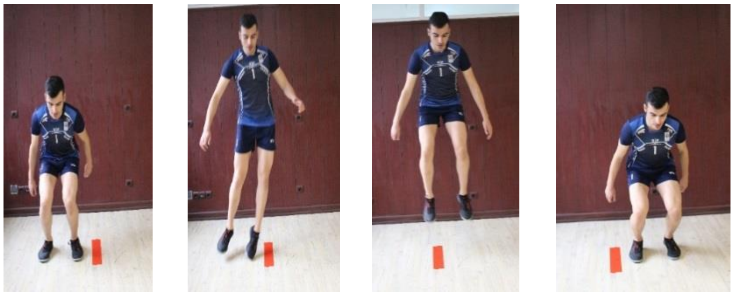

Phase 2

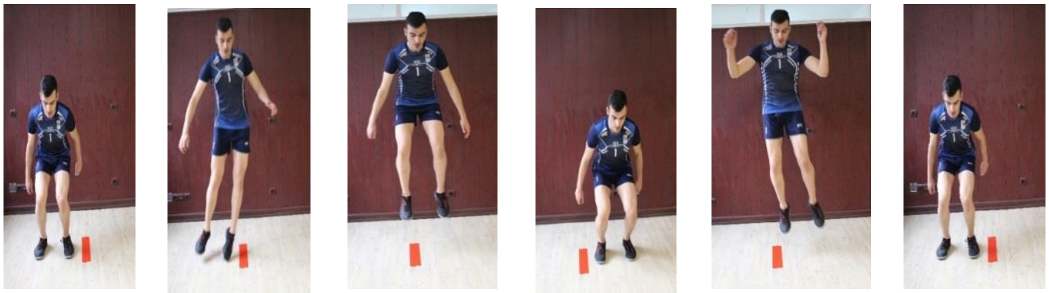

Phase 3

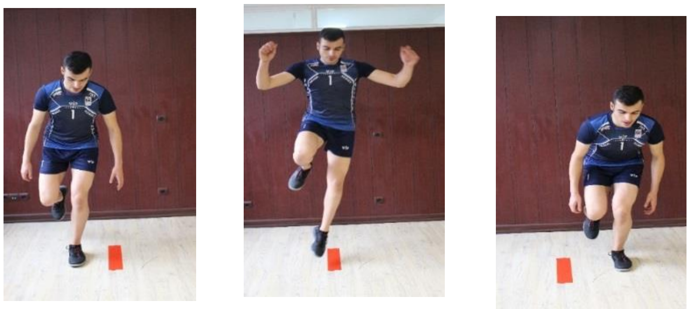

Phase 4

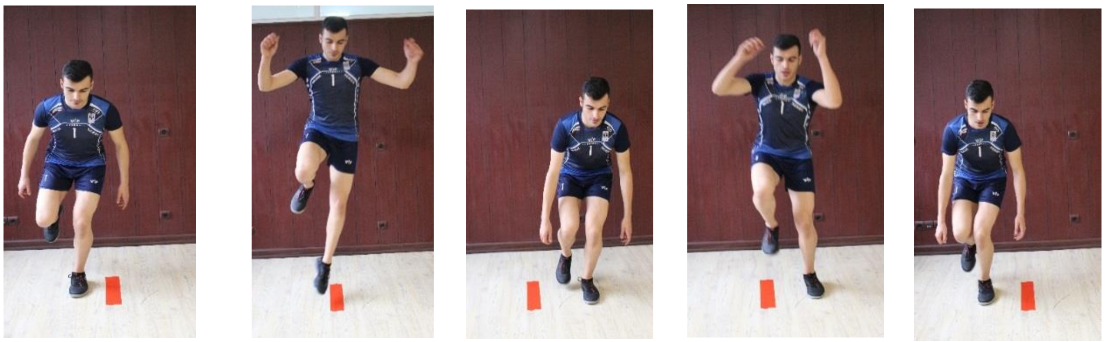

Appendix F: Lateral Trunk Progression

The trainer provides stabilization at the pelvis and lower extremities throughout the phases. The trainer instructs the athlete to bend laterally at the waist during the crunch movement and avoid non–frontal plane motion of the trunk. The athlete should also maintain the arms in a crossed position over the chest, except when involved in a partner toss-and-catch activity. Progression should be implemented when the athlete can complete the current phase with proper form and full trunk motion.

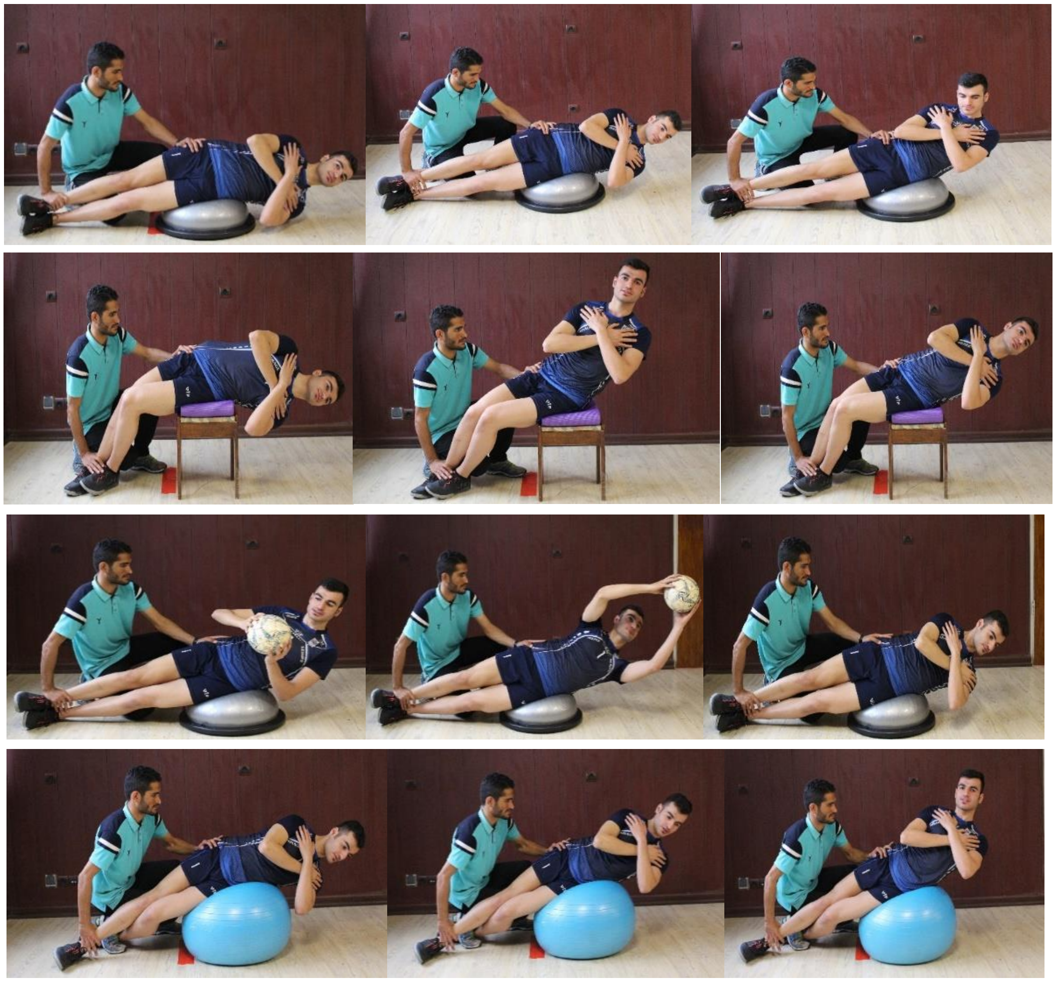

Phase 1

Phase 2

Phase 3

Phase 4

Appendix G: Prone Trunk Stability

The trainer instructs the athlete to minimize the amount of rebound (or reverberation) of the BOSU under the trunk, especially during partner perturbations. As the athlete progresses to the prone bridge position (phases 3 and 4), the 2 to 3 contact points away from the center of mass further destabilize the athlete as they alternate extremity limb positions. The goal is to avoid excessive trunk rotation and flexion or hyperextension as they lift their limbs.

Phase 1

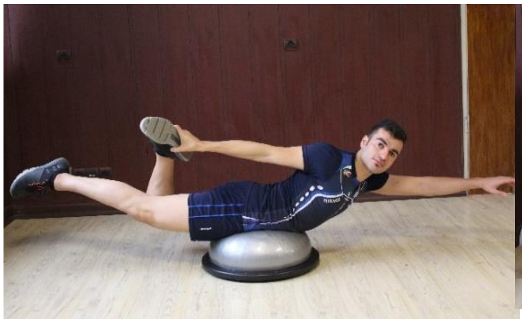

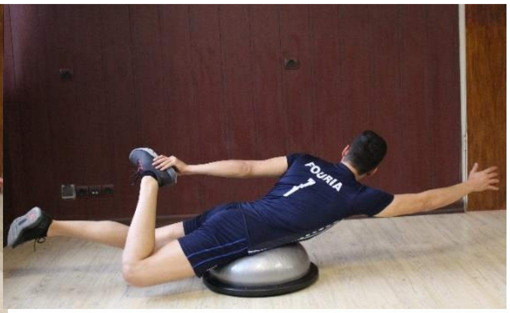

Phase 2

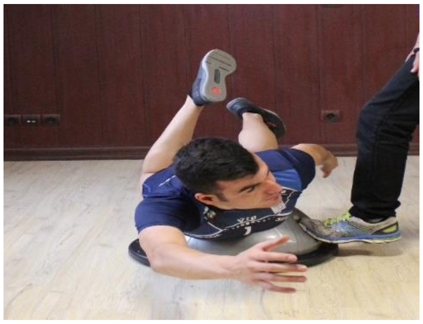

Phase 3

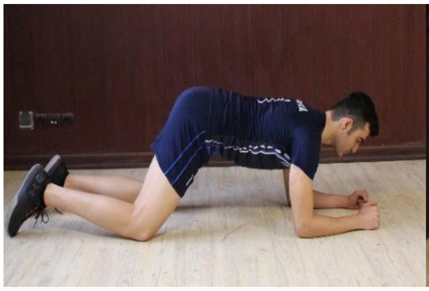

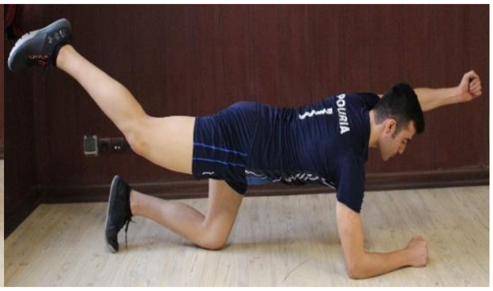

Phase 4

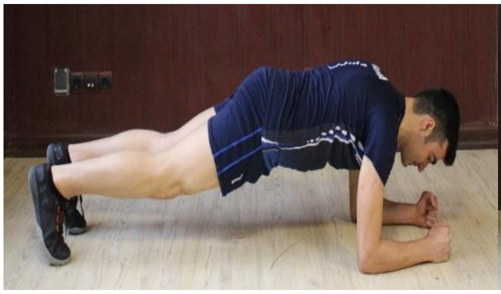

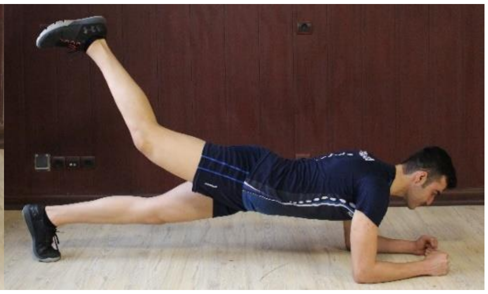

Appendix H: Kneeling Trunk Stability

The trainer instructs the athlete to maintain slight hip flexion throughout the different phases. Excessive trunk flexion and upper extremity strategy (flailing of arms) should be avoided, especially when the trainer is providing perturbations to the support surface (phase 4). The trainer should avoid administering a subsequent destabilizing perturbation prior to the athlete restoring their equilibrium.

Phase 1

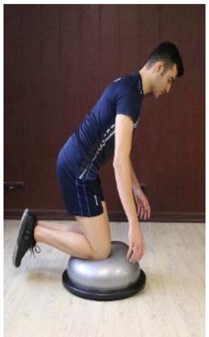

Phase 2

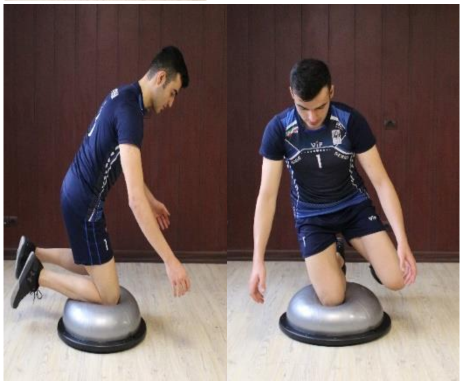

Phase 3

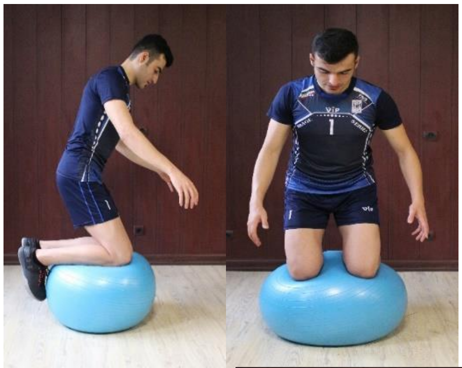

Phase 4

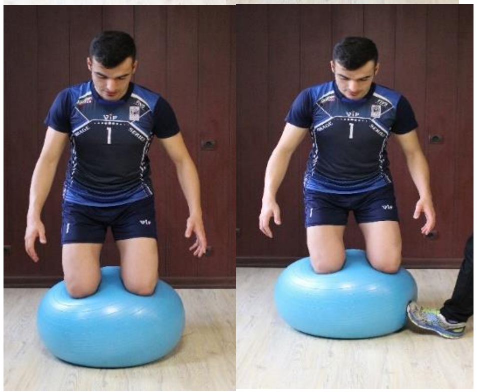

Appendix I: Posterior Chain Progression

The trainer instructs the athlete to avoid lumbar hyperextension during the bridging-task phases. Manual and verbal cues may be necessary to acclimate the athlete to a neutral pelvic position during this exercise, avoiding contralateral hip drop. As the athlete advances through stages, the goal is to perform full, uncompensated motion. Phase 3 is designed to narrow the base of support and the number of contact points to increase the difficulty of the task. In phase 4, the athlete should be instructed to minimize motion of the ball under their feet while achieving controlled hip flexion and extension.

Phase 1

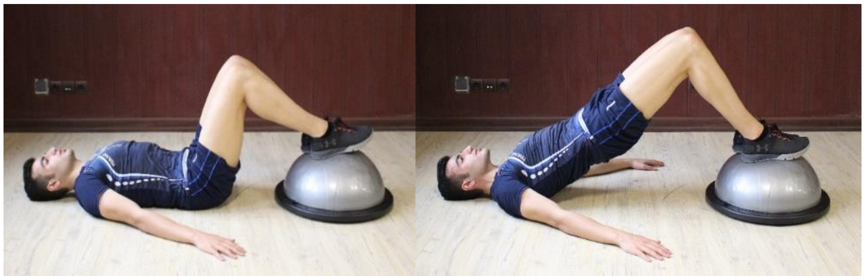

Phase 2

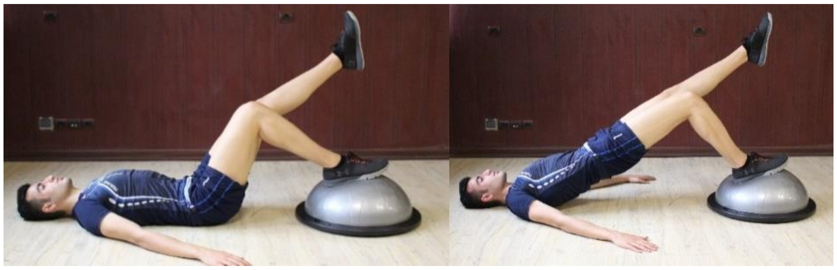

Phase 3

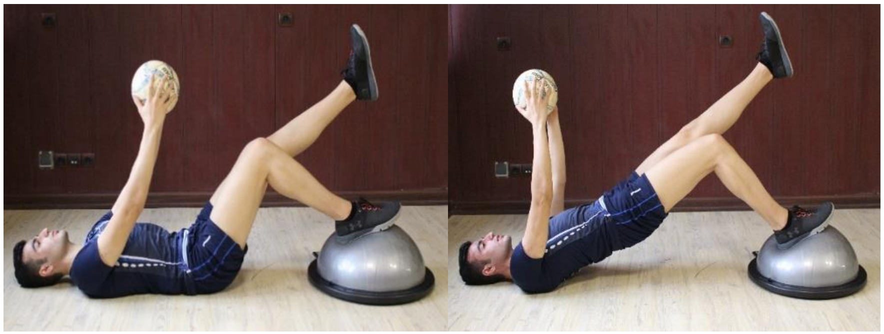

Phase 4

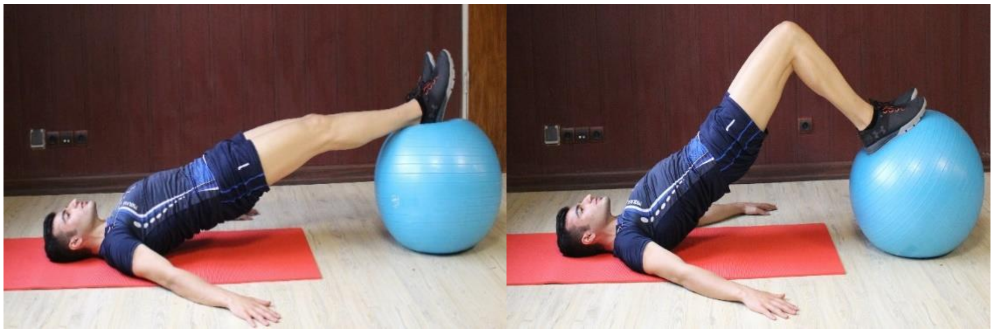

Appendix J: Romanian Dead Lift Progression

The key component to this exercise progression is the ability of the athlete to minimize trunk deviation in the frontal and transverse planes while avoiding excessive co-contraction of the muscles of the lower extremities. The trainer instructs the athlete to keep the muscles of the standing leg relaxed, with the knee slightly flexed and toes and foot relaxed. Hip hinging with an erect spine should be emphasized throughout the phases.

Phase 1

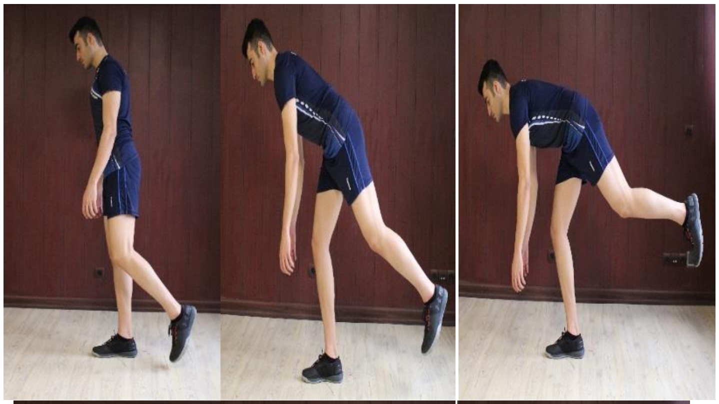

Phase 2

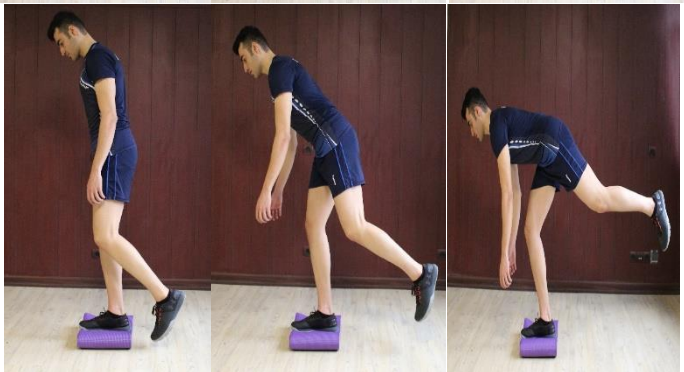

Phase 3

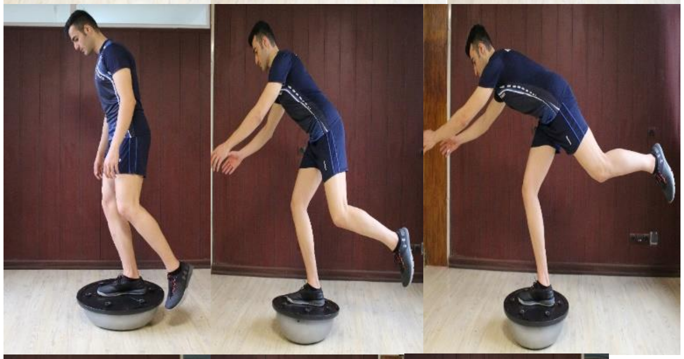

Phase 4

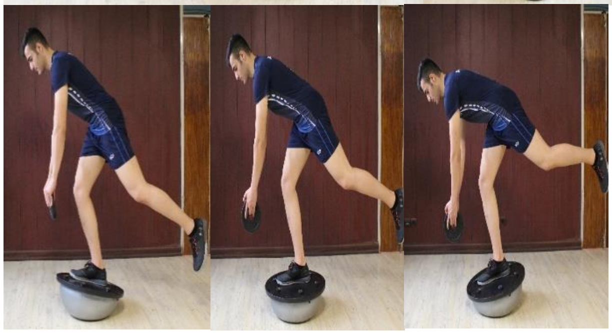

Appendix K: Lunge Jump Progression

This is a plyometric advancement of the lunge progression in appendix A, and the same emphasis should be placed on the mechanics of the lead leg and trail leg, as well as the trunk. The trainer instructs the athlete to maintain more weight toward the lead limb to generate adequate power for the jump and maintain balance. The trainer instructs the athlete to descend into a deep–knee flexion hold upon each jump take-off and landing, avoiding excessive non–sagittal plane motion of the lower extremities and trunk.

Phase 1

Phase 2

Phase 3

Phase 4

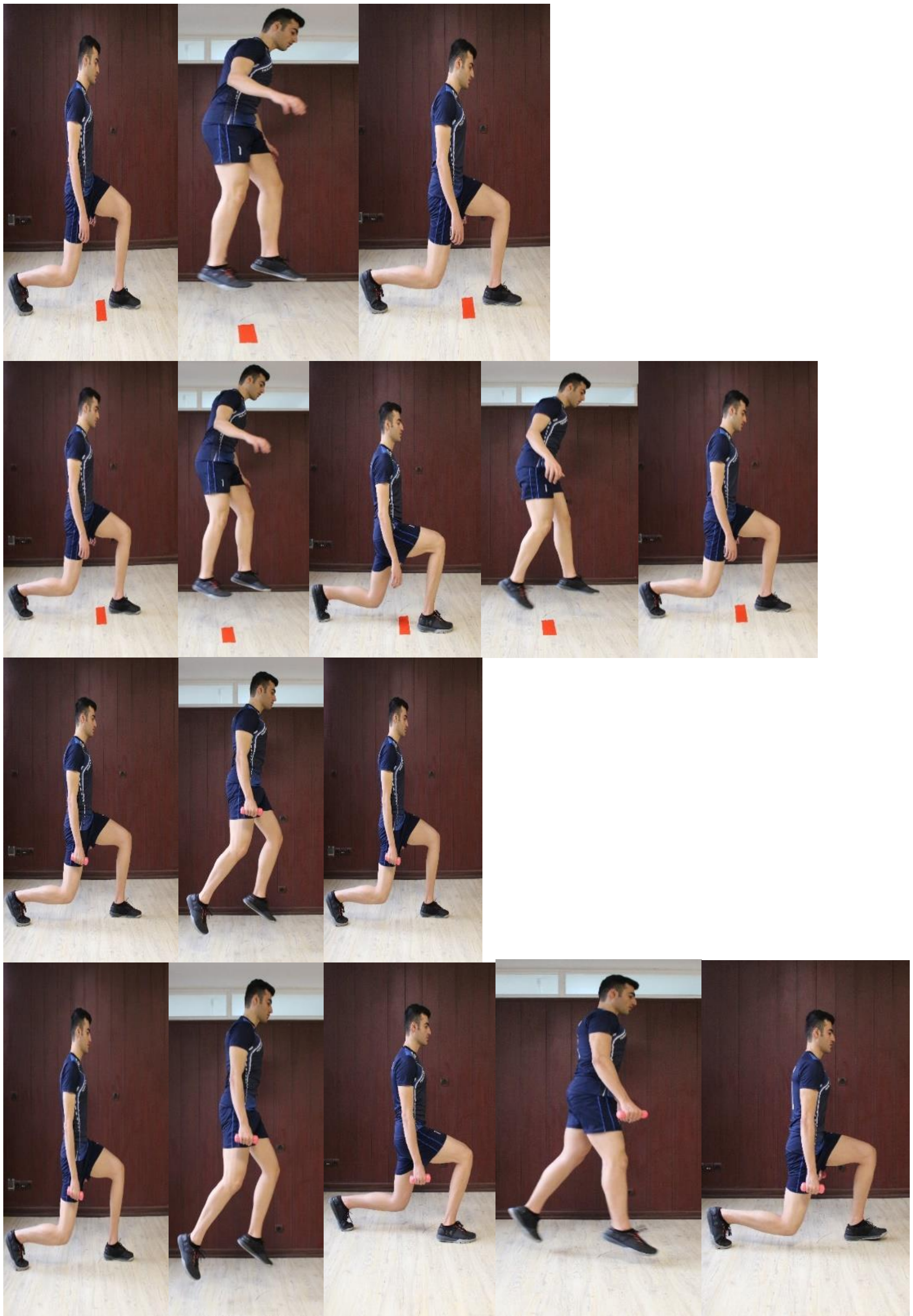

Appendix L: External Focus Instruction.

Single leg stance on unstable platform

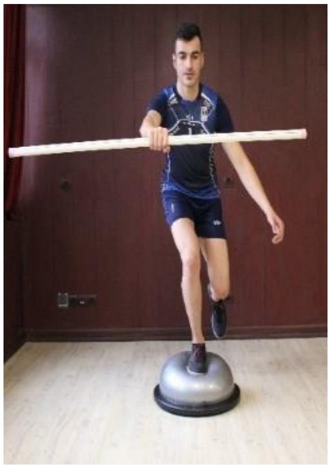

Keep the bar horizontal.

Single leg squat

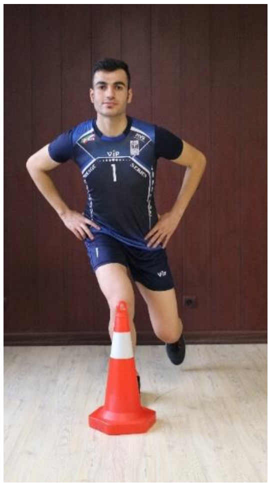

Stand on 1 leg and reach slowly towards the cone with your knee while bending your knee.

Single leg hop for distance

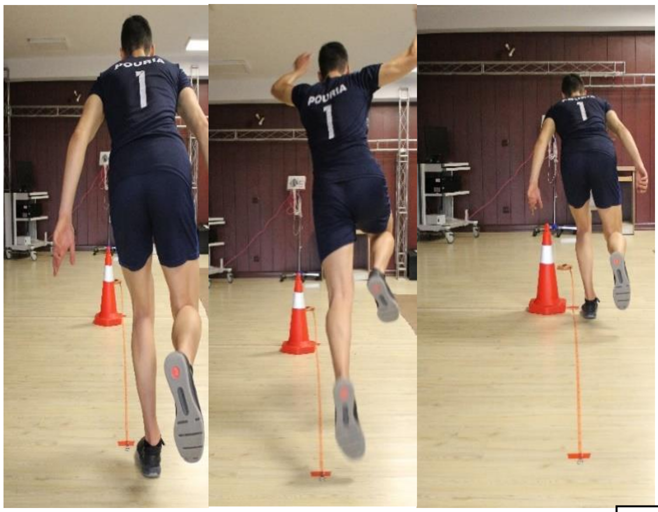

Jump as far as you can, while jumping, focus on jumping as close to the cone as possible.

(Walking) lunges

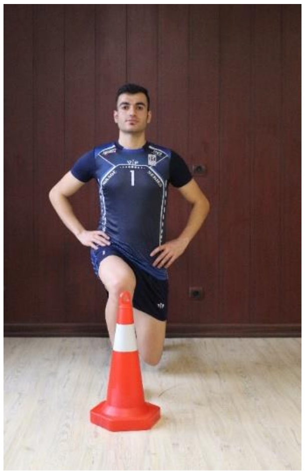

Lunge slowly at an even pace while pretending like you are having a plank on your back point your knee towards an imagery point in front of you / reach slowly towards the cone.

Appendix M: External Focus Instruction.

Double leg squat

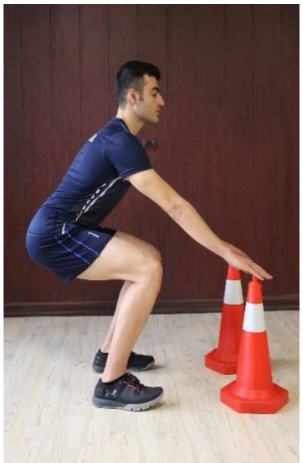

While bending your knees, reach towards the cones with your hands and point your knees towards the cones Pretend that you are going to sit on a chair while keeping a ball between your knees.

Double leg drop jump

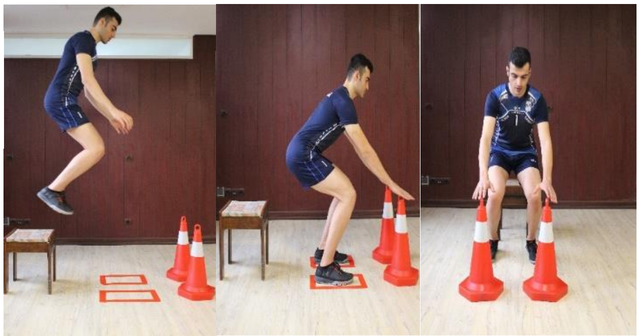

Jump down from a 30 cm box, land on the markers on the floor and put your toes and knees towards the cones.

Counter movement jump

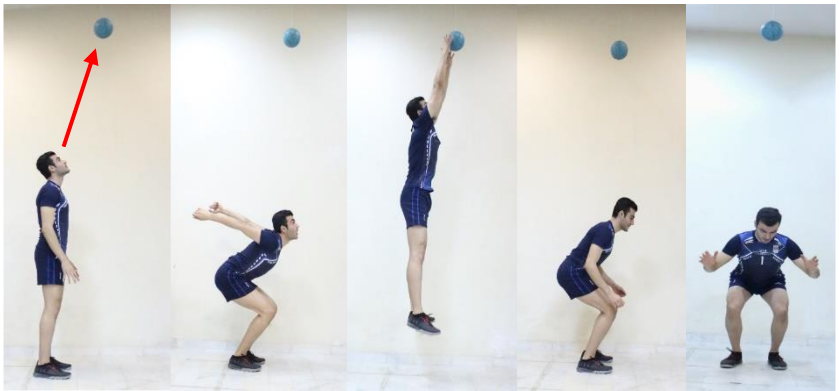

Jump as high as you can, touch the hanging ball.

Appendix N: External Focus Instruction.

Side-step cutting maneuver

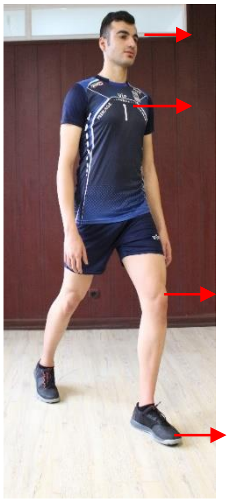

Run 4 to 5 steps straight ahead, while changing direction and making the cut, try to make a fluent motion and point your face and toes toward the direction you are going.

Vertical jump with Vertec

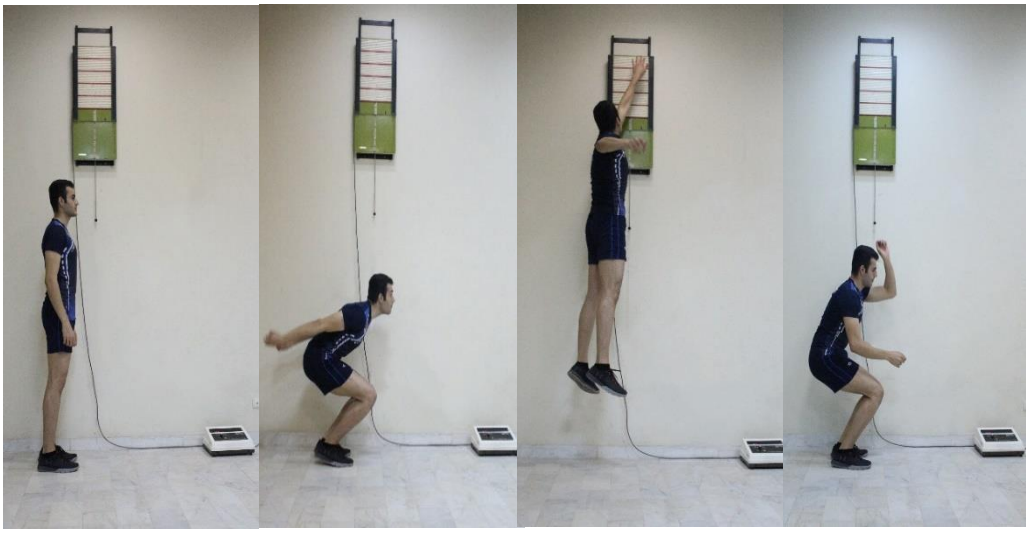

Jump as high as you can, while concentrating on the rungs of the Vertec/ball, reaching as high as possible during the jumps. Jump as high as you can, push off against the ground as forcefully as possible and pretend like you have to hold a ball between your knees.
